# The Management of Data for the Banking, Qualification, and Distribution of Induced Pluripotent Stem Cells: Lessons Learned from the European Bank for Induced Pluripotent Stem Cells

**DOI:** 10.3390/cells12232756

**Published:** 2023-12-01

**Authors:** Nancy Mah, Andreas Kurtz, Antonie Fuhr, Stefanie Seltmann, Ying Chen, Nils Bultjer, Johannes Dewender, Ayuen Lual, Rachel Steeg, Sabine C. Mueller

**Affiliations:** 1 Fraunhofer-Institute für Biomedizinische Technik (IBMT), Joseph-von-Fraunhofer Weg 1, 66280 Sulzbach, Germany; nancy.mah@ibmt.fraunhofer.de (N.M.);; 2 Berlin Institute of Health Center for Regenerative Therapies, Augustenburger Platz 1, 13353 Berlin, Germany; 3 European Collection of Authenticated Cell Cultures (ECACC), UK Health Security Agency, Porton Down, Salisbury SP4 0JG, UK; ayuen.lual@ukhsa.gov.uk; 4 Fraunhofer UK Research Ltd., Technology and Innovation Centre, 99 George St., Glasgow G1 1RD, UK

**Keywords:** stem cell data, standardization, data management, FAIRness

## Abstract

The European Bank for induced pluripotent Stem Cells (EBiSC) was established in 2014 as a non-profit project for the banking, quality control, and distribution of human iPSC lines for research around the world. EBiSC iPSCs are deposited from diverse laboratories internationally and, hence, a key activity for EBiSC is standardising not only the iPSC lines themselves but also the data associated with them. This includes enabling unique nomenclature for the cells, as well as applying uniformity to the data provided by the cell line generator versus quality control data generated by EBiSC, and providing mechanisms to share personal data in a secure and GDPR-compliant manner. A joint approach implemented by EBiSC and the human pluripotent stem cell registry (hPSCreg^®^) has provided a solution that enabled hPSCreg^®^ to improve its registration platform for iPSCs and EBiSC to have a pipeline for the import, standardisation, storage, and management of data associated with EBiSC iPSCs. In this work, we describe the experience of cell line data management for iPSC banking throughout the course of EBiSC’s development as a central European banking infrastructure and present a model for how this could be implemented by other iPSC repositories to increase the FAIRness of iPSC research globally.

## 1. Introduction

**Figure 1:**
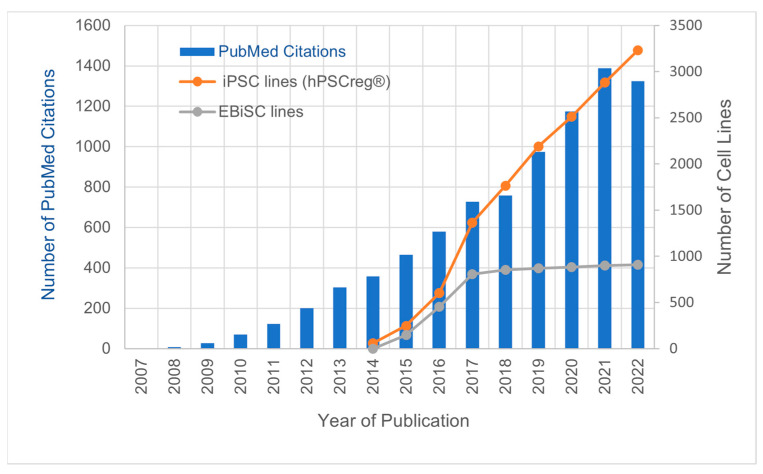
PubMed citations for the search term “induced pluripotent stem cell”, human iPSC lines registered in hPSCreg^®^, and cell lines in EBiSC, from 2007 to 2022.

Since the ground-breaking discovery of induced pluripotent stem cells in 2006 [1] research and development based on these pluripotent stem cells have steadily increased, as demonstrated by the rising number of PubMed citations since 2007 (Figure 1). Concurrently, the number of human iPSC (iPSC) lines generated worldwide is continuously growing, as evidenced, for example, by iPSC line registrations in the globally accessible registry for existing human pluripotent stem cell lines (hPSCreg^®^; https://hpscreg.eu, accessed on 13 January 2023; Figure 1). Thus, iPSCs have become indispensable research tools for modelling human development, disease modelling, and drug discovery, to name but a few applications. The rapid increase in iPSC application is driven by progress in stem cell technologies, such as easy-to-use kits, which make reprogramming widely accessible to cell biology laboratories. Advances in automation have the potential to further revolutionize downstream iPSC applications by increasing scale and reproducibility while minimizing the cost of reprogramming, expansion, and iPSC differentiation. As many academic, clinical, or institutional cell line generators are not necessarily experts in biobanking, they may not be able to guarantee reproducible quality and maintain high standards over longer periods of time. Thus, the inherent risks of local banking include the loss of samples, quality, and knowledge about relevant data. In addition, smaller projects often generate small groups of lines for internal research, which are, nevertheless, of high value for the wider community. Patients, especially those with rare or intractable diseases, are increasingly eager to donate their biological material for research, including for the generation of iPSC lines. Their main interest is to make their donated samples and derivative tools available for researchers throughout the world. Biobank infrastructures offer the prerequisite expertise, facilities, and qualified personnel to bank biological materials. Moreover, iPSC generation and local storage require the management of cells and data over years to maintain the utility of the cells, which is cost-intensive [2]. Large biobanks (Table 1) are able to implement and maintain standards, guarantee reproducible quality at scale, and, thus, mitigate the overall costs per line to make iPSC lines broadly available. 

iPSC-banking operations ensure that the cell line itself as a product and source material is banked, maintained, and quality-controlled throughout the entire supply and logistics chain, from the master cell bank to cell vials delivered to the end user. Yet, the value of human iPSCs for biomedical research lies not only with the physical cell line but includes the entire package of associated data and knowledge base behind the cell line itself (Table 2). 

The standardised and sustained management of cells needs to be reflected in the management of iPSC-associated data. Indeed, a robust quality management system, which is implemented by major biobanks and facilities, will explicitly include the management and traceability of key datasets in order to ensure that a defined quality standard is maintained. Data standardisation and provenance are essential for the successful banking and application of iPSCs in research and development, but they are faced by a number of challenges with regards to data management. An iPSC biobank must:

1. Determine which data are essential and must be generated, and hence, which data must be made available and how access is managed. This requires a set of mandatory data, which may change over time as use requirements change because of application evolution.
2. Harmonise data from different sources. Donor material or pre-reprogrammed iPSCs may be provided by diverse sources, including individual researchers and clinical research groups, university laboratories, core facilities or academic institutions, or biobanks. These may, for example, apply various informed consent models to the donors and different quality standards using variable methods to characterise the generated cells. This variability also applies to the associated datasets, with variable metadata, data, and storage formats, as well as access requirements. These data must be harmonised, missing data be generated, and all data be made findable, accessible, interoperable, and reusable.
3. Make existing inventories of iPSC lines easily discoverable to researchers. For the most part, each of the iPSC repositories in Table 1 has an online searchable catalogue of cell lines with some basic search functions to query for lines according health status, sex, and age. However, there is no minimum operational standard for data availability, a deficiency which applies to mandatory data content and formats and extends to the use of taxonomies, ontology terms, or standard interoperable identifiers (names). These are necessary to solve complex queries from users. For example, from a user perspective, is it possible to compose a genetically diverse, sex-balanced, neurodegenerative disease cohort of gene-edited isogenic cell lines for an organoid-based drug screen, ensuring all iPSCs come with the essential information needed to develop a commercial product or service? Such a query may also require interoperability across cell banks. Different approaches for stem cell data standardisation are emanating from various stakeholders [3,4,5]. However, there is no consensus yet from the stem cell community on how to achieve full interoperability, which would allow complex queries such as in the previous example.

Solutions to these challenges need to be found by all iPSC-banking infrastructures, regardless of their size. Here, the solutions and management procedures developed by the European Bank for induced pluripotent Stem Cells (EBiSC) are described and discussed.

## 2. EBiSC—The European Bank for Induced Pluripotent Stem Cells

EBiSC is a collaborative not-for-profit centralised iPSC repository, which collects iPSC lines generated by diverse laboratories worldwide and makes them, plus iPSCs predifferentiated to defined cell types, available centrally and worldwide (with them having been distributed to >36 countries across five continents so far) under single transfer agreements for research use. The first phase of EBiSC was supported by the Innovative Medicines Initiative (IMI) programme as a joint initiative between the European Commission and the European Federation of Pharmaceutical Industries and Associations (EFPIA), with the aim of establishing a quality iPSC resource that could be used by academia and pharma alike to develop new iPSC-based applications. Building upon the success of its precursor (EBiSC), the EBiSC2 project secured a second round of funding from the IMI2 towards a self-sustainable iPSC bank, including the development and provision of predifferentiated cells and services. EBiSC is now developing as a sustainable long-term, independent, not-for-profit biobank. It currently holds 928 iPSC lines as of February 2023. When EBiSC was established in 2014 [6,7,8], the project liaised with an established data management platform dedicated to human pluripotent stem cells, the human pluripotent stem cell registry (hPSCreg^®^; https://hpscreg.eu, accessed on 1 February 2023). This mutually beneficial arrangement allowed EBiSC to benefit from the data standards already established in hPSCreg^®^ [9,10,11] and promoted the refinement of hPSCreg^®^ with regard to the requirements for iPSC generation and use so that iPSCs could be better accommodated in the established registry. Here, we present the data infrastructure that EBiSC has established with hPSCreg^®^ as a general paradigm for iPSC bank sustainability. 

## 3. EBiSC Data Management Infrastructure

**Figure 2:**
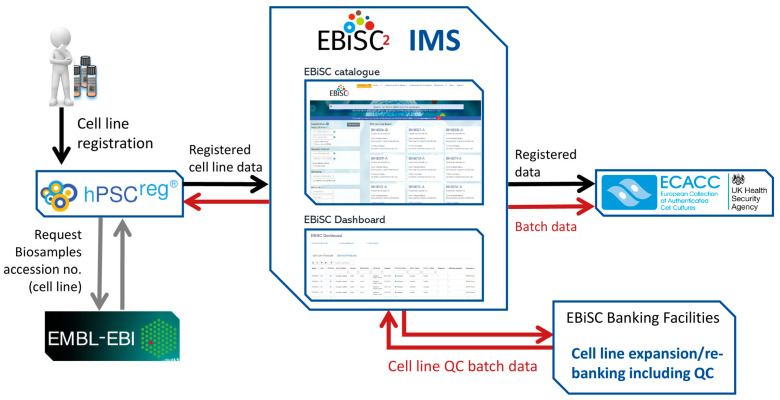
Graphical overview of the EBiSC data flows. The EBiSC Information Management System is the heart of cell product data management (IMS). Depositor-provided cell line data are shown in black arrows. Data generated within EBiSC, including QC batch data, are shown by red arrows. Grey arrows to EMBL-EBI depict requests to obtain Biosamples accession numbers for registered cell lines and donors.

The data management infrastructure was set up to centrally collect iPSC-associated data throughout all aspects of biobanking, including sample collection, cell line manufacturing, cell quality control, banking, and distribution (Figure 2) [6]. EBiSC integrates three major infrastructures, including the hPSCreg^®^ platform, the EBiSC Information Management System (IMS), and the EBiSC catalogue (https://ebisc.org/search). Moreover, EBiSC uses services from external data resources, such as the European Bioinformatics Institute (EBI) and the European Genome Phenome Archive (Table 3).

### 3.1. Fair Data Principles

FAIR data principles are borne out of the ease with which technologies enable researchers to generate “big data” and necessitates sensible data management to ensure research results are findable, accessible, interoperable and reusable (FAIR) [12]. In its purest form, the full implementation of the FAIR data principles would make data machine-readable and machine-actionable; thus, not only would one be able to locate specific data across diverse resources, but machines would also be able to perform data discovery, integration, and analysis on a much wider scale than a human operator alone could accomplish with a series of manual, error-prone steps. In practice, EBiSC has taken several steps to promote the FAIRification of pluripotent stem cell data, which are outlined in Table 4 and described further below.

### 3.2. Identifier and Labelling

**Figure 3:**
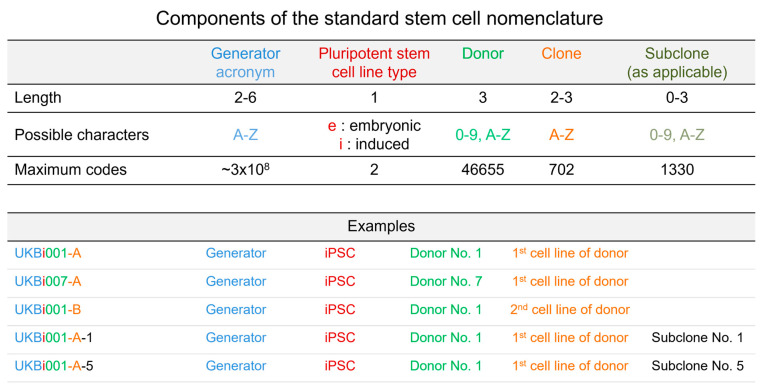
Unique identifiers for hPSC lines registered in hPSCreg^®^. The standard stem cell nomenclature is composed of five parts: the generator, pluripotent stem cell line type, donor, clone, and subclone. In this schema, a generating institution can specify embryonic or induced pluripotent stem cell lines from multiple cell lines from a single donor. Genetically modified versions of existing (parental) cell lines are designated as subclones with an additional suffix. In the first example, UKBi001-A indicates an iPSC line from generator Universitätsklinikum Bonn (UKB), from the first line derived from the donor number 1. From the same generator, the second example represents the first-made iPSC line from donor number 7. The third example defines the second iPSC line generated by UKB from donor number 1. The following examples are also all generated by UKB from donor number 1 and represent different subclones (number 1 and 5).

The assignment of a unique persistent identifier for each cell line is key to allow the unambiguous identification of iPSC lines, not only within the bank but also worldwide. The persistent traceability and discrimination of iPSC lines and their relationships in terms of source cells, donor association, or available genetically modified subclones are essential for an iPSC biobank with long-term storage and distribution capacity. Using hPSCreg^®^ as its initial data entry point, an application programming interface (API) assigns each cell line a unique identifier according to a standard stem cell nomenclature even before its deposition in EBiSC (Figure 3) [13]. This identifier is registered and publicly accessible, thus preventing reuse and making it persistent and unique globally. This uniqueness is the basis for the interoperability of all data associated with the banked iPSC lines, whether these are generated at EBiSC or anywhere in the world, thereby amplifying the cell lines’ specific knowledge space and facilitating stakeholders’ valuation of research results. In EBiSC, different batches generated from one specific cell line and comprising several vials are not treated as separate cell lines and, therefore, receive unique identifiers of the form *<cell line identifier>_<batch>_<vial number>* to allow the detailed batch and vial tracking of this specific cell line. In addition and parallel to a unique cell line identifier, Biosamples accession numbers are assigned to each iPSC line and its donor (https://www.ebi.ac.uk/biosamples/) [14], thereby facilitating the direct linkage of any deposited data at EBI. These linked datasets may include de-identified data, which can be made freely available, such as processed transcriptome values [15], or sensitive data like genetic or clinical data, which can be made available subject to managed access through EGA [16]. The accession numbers are issued and shared between three major international biological data repositories, namely EMBL-EBI (Europe), NCBI (US), and DDBJ (Japan). Moreover, all EBiSC lines are linked via their unique identifier to the Research Resources Identifier (RRID) [17].

Finally, the use of these identifiers strips any direct link from the cell lines to the actual donor as the identifiers are anonymous. Any link to the donor, if it exists, remains the responsibility of the cell line generator or clinical contact. Therefore, the anonymised cell line and donor IDs used by EBiSC serve to protect the cell line donor in accordance with the General Data Protection Regulation (GDPR; https://gdpr-info.eu/) and equivalent data protection legislations, which are the applicable legal frameworks for EBiSC data operations.

### 3.3. Data Types

Data associated with iPSC lines are provided by the depositor upon their initial registration via the hPSCreg^®^ platform and further verified and enriched by EBiSC during the expansion, banking, and quality control (QC) processes. The data types and categories are summarized in Table 2 and described below. 

The first category of data relates to the iPSCs and their banking. This is a “basic” dataset that allows for research use by including donor demographics, age (range) at the time of sample donation, sex, disease status and phenotypes, as well as information on the reprogramming method, culture conditions, and gene editing (if applicable). The EBiSC online catalogue (https://ebisc.org/) shares these data to users to support cell line selection and use (Table 5). Additional data elements, which are crucial to EBiSC as a cell-banking and distribution operation, include data on cell line quality, for example, short tandem repeats (STRs) for authentication, karyotypes, and adventitious agent testing results. As a central iPSC hub, EBiSC also records a trail of detailed information on the cell line’s culturing history, including media, passaging reagents, plate coatings, and all associated protocols, such that the “handling provenance” of the cell line is known. The qualification of the cell lines for distribution is carried out for the master cell bank and for each batch generated by EBiSC, including strict batch release testing [18]. These batch-specific data are stored in the EBiSC Information Management System (IMS). The data are summarized, documented, and stored in EBiSC, and a subset of data relevant for general cell line information (e.g., the reprogramming method, genetic modifications, etc.) and batch-specific data (e.g., QC release data and culture recommendations) are detailed on a public certificate of analysis (CoA), which is stored in the IMS.

A second category of data is supplementary data to add value to the cell line package as a whole, along with the critical and mandatory data related to the EBiSC lines. The availability of such data is assessed at the time of cell line deposition and include, for example, information on differentiation bias or the detailed clinical, genetic, or epigenetic data of the donor or cell line (e.g., sequencing or SNP genotyping arrays). EBiSC encourages the original depositors to make these data known and accessible to end users in concordance with existing data privacy regulations. These supplementary data may be held directly by EBiSC or deposited to an external repository such as the European Genome Phenome Archive (EGA). In any case, access to sensitive personal datasets is managed by the EBiSC Data Access Committee (DAC) (see the legal framework below).

A third category of data relates to sales of its products and the tracking of consumer trends. These data are used to analyse the historical sales statistics of EBiSC lines and appropriately align cell line parameters such as certain diseases. These data are relevant for the long-term sustainability of the EBiSC bank and its not-for-profit character.

Specific data on worldwide shipments of EBiSC cell lines, such as customer order details, are not stored in the IMS but in a separate data protection-conforming management system.

### 3.4. Catalogue Search and Filter Functionality

The EBiSC catalogue (https://ebisc.org/search) fed by the EBiSC IMS has different entry points to enable cell line discovery: (1) general full-text search; (2) the filtering of cell lines via a select list of data elements; (3) predefined cell line collections, including relevant controls (https://ebisc.org/collections); (4) a list of cell lines with similar properties like the cell line the user is currently viewing, for example, if this line is out of stock. These functionalities aim to ease finding cell lines of interest in a rapidly growing and diversifying collection and are steadily extended to adapt to user needs.

General full-text search: a query will be searched against a list of terms, including cell line IDs, genes, diseases, Biosamples accession numbers, and any free-text fields (public notes, karyotypes, or functional data fields). This non-structured search method identifies lines with specific comments in the free text; for example, searching for “duplication” yields a list of cell lines with some form of a genomic duplication. 

**Figure 4:**
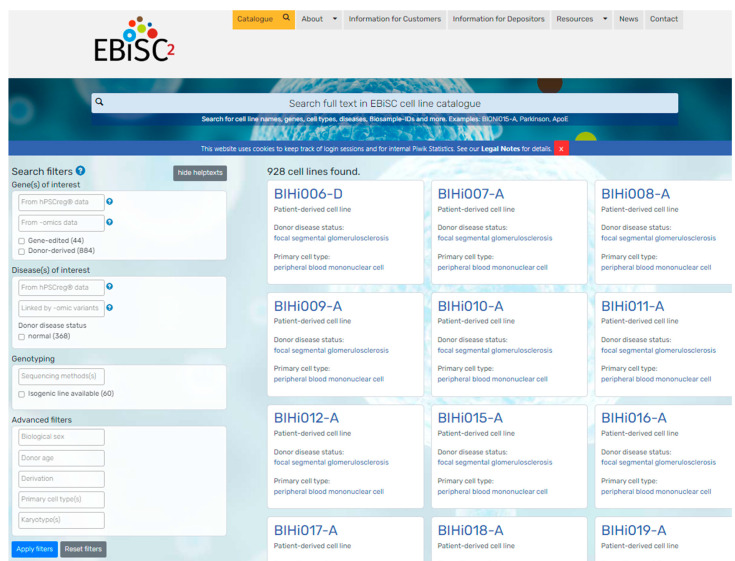
EBiSC search interface. Snapshot of the EBiSC catalogue search interface is shown, with search filters in the left sidebar and a list of search results in gallery mode on the right side.

Filters: The second way to find cell lines of interest is to filter for lines based on discrete terms that have been collected and recorded using controlled vocabularies (e.g., official gene symbols) or ontologies (e.g., disease ontologies such as MONDO, DOID, or ORDO; cell ontology (CL) for cell types) [19,20,21,22]. For information that currently cannot be described with an ontology or taxonomy term, a drop-down list of terms is provided to ensure consistency in recorded information (e.g., cell line-reprogramming methods). EBiSC cell lines can be filtered (Figure 4) by disease (donor disease or disease associated with a gene edit); genes of interest (genes with verified genetic variants or variants detected via single-nucleotide polymorphism (SNP) array genotyping); the availability of linked datasets such as omics data, derivation methods, karyotyping results, and source cell type for reprogramming; and donor characteristics such as sex and age. The use of controlled vocabularies has facilitated the implementation of filters for searching for both genes and diseases. However, some data elements, such as karyotyping, are currently saved as free-text fields due to the lack of suitable standard formats for recording these data. In these cases, the manual curation of the free-text data has been completed to enable filtering based on discrete, defined categories. For ease of browsing, the direct filtering of cell lines associated to diseases is enabled by simply typing the query disease into the URL. For example, lines associated with Alzheimer disease can be found at https://ebisc.org/search?q=&hpscreg-diseases=%5B%22Alzheimer+disease%22%50D.

Predefined cell line collections: EBiSC hosts a number of cell line collections curated for a specific research purpose. For example, there are currently five collections of isogenic lines with genetic modifications aimed at studying different aspects of neurodegenerative diseases. The genetic modifications include edits to repair or insert disease-related genotypes, the insertion of reporter genes, or the inclusion of inducible transgenes to modulate cell differentiation. The available collections are highlighted on a separate web page for easy access (https://ebisc.org/collections).

## 4. Ethical and Legal Framework

The overall goal of EBiSC regarding this framework is to ensure operational trust for the use of EBiSC iPSC lines and transparency about ethical and legal permissions and restrictions. The EBiSC bank tries to ensure from the outset that all processes, from tissue donation to the generation of iPSC lines, are compatible for the centralised storage and global distribution of iPSCs by commercial and non-commercial entities for research purposes. From a data management perspective, critical information is stored and managed, such that there is a clear ethical provenance of the source biomaterial, and there are clear provisions to protect sensitive donor data while allowing research use. Moreover, third-party obligations (TPOs) associated with the iPSC line must be permissive for research purposes, and these submitter-defined TPOs are documented in end-user agreements and made transparent and understandable to the prospective end user.

### 4.1. Informed Consent Data 

Consent documentation is mandatory for cell line deposition in EBiSC. Prior to cell line deposition in the EBiSC bank, documentation surrounding the ethical provenance of the iPSC line is deposited and reviewed. These essential documents include templates of the participant information sheet and informed consent forms, information on the ethical review board that reviewed the consent materials, and its positive opinion and decision reference number. Additional documentation used in the consent process, such as material used where consent is sought from children or adults with reduced capacity, is also reviewed. Furthermore, EBiSC internally stores a paper trail documenting the review process, including the EBiSC consent review, letters from ethics committees (IRBs), and the consent verification statements, which are written statements from the cell line provider affirming that the donors have signed the informed consent forms, and the donor’s replies to any optional clauses in the consent forms are disclosed to EBiSC for appropriate recording. As a whole, the documentation trail constitutes a complete data package to document a transparent, voluntary, and informed consent process. Proof of the consenting process is absolutely required to provide a legal basis for the downstream use of the donated biological material. The consent documentation is reviewed for a checklist (Table 6), and the corresponding information is saved in a structured questionnaire during the initial cell line registration to ease automated analysis. 

The assessment of third-party obligations (TPOs) is required as the EBiSC collection banks lines from diverse projects, which may have specific conditions attached to the re-use of resources generated within these projects. Such terms may include, for example, a research restriction to a specific disease area, such as neurodegenerative diseases. Moreover, processes or reagents employed to generate the iPSC line may have downstream implications for the end users of the cell lines as these processes or reagents may be associated with intellectual property (IP) rights. Licensing agreements associated with any IP must be documented. These may include IP associated with reprogramming (e.g., iPS Academia Japan license) or gene editing, such as the use of CRISPR/Cas or the introduction of reporter gene constructs containing fluorescent dyes. 

Once the consent documentation and TPOs associated with the cell line are deemed sufficient for EBiSC banking, the cell line owner will complete the EBiSC Material Deposit Agreement (EMDA), which includes statements on how data can be used according to GDPR and any associated TPOs. EBiSC validates the EDMA and adds relevant TPOs to the corresponding cell line information pack (CLIP), which delineates all terms of use specific to the cell line. Together, these documents set the required legal framework for EBiSC operations. A similar process is employed for EBiSC iPSC-derived cells with TPOs and any relevant use restrictions recorded in a Product Information Pack (PIP). 

### 4.2. Downstream Usage Agreements

Customers who purchase EBiSC cell lines are subject to the general terms of the EBiSC Access and Use Agreement (EAUA), along with the conditions collated in the CLIP or PIP (Table 7). The EAUA is a formal legal agreement between the end user (researcher) who will work with the cell lines or data and EBiSC. The data associated with cell lines are generally accessible from EBiSC, even without purchasing a cell line. General characterisation data are openly accessible from the online catalogue, and there is no explicit license associated with data usage. However, sensitive personal data, such as genetic data or clinical data, are available to the research community upon a managed access protocol. As these kinds of data require specific protection under the General Data Protection Regulation (GDPR; https://gdpr.eu/), implemented measures ensure that the research community is able to access and use the data for research. The access to sensitive datasets is managed by the EBiSC Data Access Committee (DAC), which reviews data access requests from interested researchers and grants access, contingent primarily upon (i) the planned research activity and experience of named personnel and (ii) clashes with any defined use restrictions. For example, any research that is restricted by informed consent as outlined in the CLIP, or any attempt to re-identify donors, would not be permitted. An authorised legal representative of the researcher’s institution is required as a signatory for the EBiSC Data Access Agreement (DAA). 

## 5. Discussion and Outlook

Induced pluripotent stem cell lines are valuable tools for disease research as a renewable starting material for differentiation into organoids or specific cell lineages, with the potential for innovative applications in disease modelling, drug development, or cell therapy. A large proportion of the generated cell lines and established banking facilities have been made possible by high-level, government-funded research programmes and large private enterprises (Table 1). These infrastructures are complemented by cell line collections driven by local or federated initiatives (e.g., PluriCore and COREdinates [2,23,24]). Without exception, these programmes and institutes have the same goal: to make the iPSC lines widely available for the advancement of scientific research and the development of novel therapies. To address this goal, the EBiSC bank was established to make iPSC lines accessible to academic and for-profit entities, with advanced characterisation status, clear usage agreements, and freedom to operate. 

To build up a diverse and rich resource for quality-controlled iPSCs, EBiSC acquired iPSCs from multiple sources, including individual researchers, other iPSC banks, or established disease-specific cohorts, and generated cells within the EBiSC partnership. EBiSC is additionally developing protocols for the distribution of predifferentiated cells representing multiple lineages and cell types. This diversity of sources and depositors creates challenges with regards to quality assessment, the harmonisation of documents, and data management. The human pluripotent stem cell registry platform (hPSCreg^®^; https://hpscreg.eu) manages pluripotent stem cell data from global providers and, thus, developed solutions for managing hugely diverse data sources, documents, and formats. As the hPSCreg^®^ is a public resource, its platform is available for broad application and partnerships. Thus, EBiSC and hPSCreg^®^ jointly built a model and platform to enable the mass standardisation and dissemination of key data points to support research. This allowed EBiSC, for example, to use the hPSCreg^®^ API to generate persistent unique cell line identifiers, to apply established standardisation tools for data annotation, and to adopt the implemented characterisation matrix of structured data, including ethics-related information and the advanced FAIRification setup of iPSC data. 

To fit the specific multi-partnership structure and the physical banking purpose of EBiSC, additional data management tools were implemented to handle data generated during batch manufacturing and quality control, sales and customer interaction, legal and ethical framework operations, as well as data exchange between partners. These are managed by the EBiSC IMS (Figure 2), which coordinates, exports, and transports data within EBiSC, for example to populate and update the EBiSC catalogue or to monitor batch and sales status. Moreover, EBiSC further enriches the data of its iPSCs by scouting and linking external resources for supplementary annotations, publications, and applications, generating an iPSC line-centred knowledge hub.

The EBiSC bank currently contains >900 iPSC lines and is continually growing. Globally, many more iPSC lines are available, and their number is increasing daily. However, without deposition into a public repository, the existence of these iPSCs is often unknown to the community, and even if published, the use of ambiguous identifiers often makes it difficult to locate lines and associated data beyond a small group of knowledgeable peers. Locating cell lines for specific research purposes is further complicated by the existence of many separated, individual catalogues developed for a specific cell collection with limited exchange options and low data interoperability between iPSC banks. The trend towards the establishment of multiple smaller iPSC resources, for example dedicated to certain diseases, applications, or localities, further adds urgency to developing platforms that allow the interoperability, exchange, and linking of data. EBiSC has set up a basis to enable this by using standards as much as possible and by using a global open access platform for its data deposition. 

Allowing researchers to perform detailed cell data queries over many resources is currently a time-consuming manual task. For example, the composition of an iPSC collection with specific features for drug screening is a laborious, time-consuming task, not taking into account clarifying the ethical provenance and usage restrictions for all iPSCs in the collection. From a data perspective, dedicated pluripotent stem cell data discovery tools have been developed with the aim of making PSC data more accessible to the research community, independently of whether or not cell lines have been banked in a recognized cell repository. Eagle-i-net [25] focussed on creating a federated network of iPSC resources in the US, while the integrated collection of stem cell bank data (ICSCB) [26] gathered data from five major hPSC resources (databases: eagle-i-net, SKIP, and hPSCreg^®^; cell bank catalogues: CIRM and BRC RIKEN) into one collection, making it possible to query all five resources at once. Unfortunately, some of these databases are no longer current due to a lack of funding (ICSCB and SKIP) or are completely defunct (eagle-i-net). How to link scattered stem cell-based data over multiple resources is also a problem in the advanced cell therapy field, where stem cell data are scattered among multiple resources [27]. Currently, to the best of our knowledge, hPSCreg^®^ is the only active, independent global stem cell data resource accepting registrations from around the world, regardless of the cell line distributor. hPSCreg^®^ has been funded since 2007 by the European Commission (EC) as a registry to keep track of pluripotent stem cell research and enforce the ethical provenance and biological quality of hPSC lines used in EC-funded research projects. To perform this function for the EC and the global stem cell community, hPSCreg^®^ collects a large body of hPSC-associated data, encompassing key information about the cell line donor, the ethics, and the biological properties of the cell line (pluripotency and gene edits). All data are publicly available through a persistent unique identifier, which is based on a standard nomenclature. EBiSC recognised these benefits at its beginning and joined forces with hPSCreg^®^ for EBiSCs’ data management to establish a gold standard of sustainable data management in PSC biobanking, which also serves as a model to other resources and banks. Adopting the data standards and data template developed by hPSCreg^®^ and implemented by EBiSC provides a practical model to structure the iPSC data landscape with immediate effects on the interoperability, information exchangeability, and linkage of data on biological characterization or ethical provenance.

To further enrich cell line data towards a knowledge network, data linkage and scouting must go beyond iPSCs themselves but also extend to the rich body of omics data generated, application in standardised and reproducible assays, preclinical and clinical work, cohort-based drug screening, and other upcoming utilisations. This knowledge network would not only provide researchers and industry with an enriched matrix to base experiments on, but it would also inform resources such as EBiSC as to which areas need resource investment, enabling it to tailor its collection and promote cell lines in their possession.

Similarly, understanding and navigating the legal and intellectual property landscape, with regard to restrictions on the use of iPSC lines, require complex knowledge. EBiSC has developed an iPSC-focussed legal framework, which allows extension to further application areas such as the development and distribution of predifferentiated cells. However, this task is complicated by the global diversity of regulatory and legal frameworks in which iPSC banks, and especially their customers, need to navigate. This situation grossly hampers the utility of existing resources and forces users to develop their own cell line or rely on a few legally well-documented lines instead.

Not only is the generation of ever-increasing numbers of iPSC lines in countless laboratories a waste of resources in many cases, as equivalent lines are unknowingly already available, but they are also not findable. Moreover, it creates uncertainties with regards to quality assurance, ethical provenance, and legal freedom to operate and limits efforts towards reproducibility. This lack of reliable information and quality creates risks for funders and researchers as the reproducibility of generated results may be low just because of the uncertainties associated with the used source material, or primary errors may amplify in derived cell types. These risks are especially impactful for preclinical work or assays used in industrial application, for example, in drug screenings. Here, banks operating under strict quality control testing regarding biological, ethical, and legal parameters lower these risks significantly. The strict application of international guidelines and standards for cell lines and data, such as those in development by the International Society for Stem Cell Research (ISSCR) and International Standards Organisation (ISO), will further promote the reproducibility of research. 

Finally, making the iPSC-associated data FAIR will not only advance access to the physical cell lines themselves but will also provide data on cell lines, which, because of their structured representation, can be used to virtually validate and compare research results obtained with other cell lines. The inclusion of further data areas and annotations will make this virtual modelling feature increasingly useful. Further improvements in FAIRification and interoperability towards machine readability are ongoing in hPSCreg^®^, and this will in turn substantially improve the discoverability of EBiSC cell lines through better search mechanisms. 

In conclusion, EBiSC established a data management platform to manage iPSCs and associated data from global cell line depositors. It provides a service to sustainably maintain iPSC stocks for the global community and make these available. This ability extends to the data associated to the deposited cell lines. Thus, EBiSC also provides, via the hPSCreg^®^ platform, a permanent safe haven for iPSC-associated data for laboratories worldwide, which are, at the same time, globally accessible. Moreover, the presented EBiSC–hPSCreg^®^ paradigm is a model for iPSC banks to connect to an existing stem cell data infrastructure, such as hPSCreg^®^, for sustainable stem cell data management. Data services could be further expanded towards providing freedom-to-operate analysis, taking regulatory issues into consideration, and providing matchmaking services to connect iPSC line suppliers and parties interested in prequalified lines for selected applications.

## Figures and Tables

**Table 1:** Brief overview of some of the largest important human iPSC biobanks with worldwide distribution capacity.

iPSC Bank	No. of Human iPSC Lines in Catalogue (February 2023)	Description
BRC RIKEN ^1^	4239	Contains lines from healthy/disease donors; holdings from primarily Japanese public institutions
WiCell ^2^	1447	Maintains collections of healthy/disease and gene-edited iPSC lines
Fujifilm CDI ^3^	1554	Hosts the California Institute for Regenerative Medicine (CIRM) and other collections
Sampled ^4^	479	Catalogue includes the National Institute of Neurological Disorders and Stroke (NINDS; 184 lines) and the National Institute of Mental Health Repository (NIMH) and Genetics Resource (NRGR; 295 lines)
Coriell Institute for Medical Research ^5^	206	Distributor for various collections, including the Allen Institute Cell Catalog (56 fluorescently tagged iPSC lines); National Institute of General Medical Sciences (NIGMS; 108 lines); Orphan Disease Center (ODC; 9 lines); and National Aging Institute (NIA; 33 lines)
EBiSC ^6^	928	Diverse collection of healthy/disease iPSC lines produced from EC- and UK-funded projects

1. https://cell.brc.riken.jp/en/ (accessed on 28 December 2022). 2. https://www.wicell.org/ (accessed on 21 December 2022). 3. https://www.fujifilmcdi.com/ (accessed on 21 December 2022). 4. https://sampled.com/ (accessed on 22 December 2022). 5. https://www.coriell.org/ (accessed on 22 December 2022). 6. https://ebisc.org/ (accessed on 20 February 2023).

**Table 2:** Cell line data checklist for an iPSC-banking operation.

Data Area	Data Types	Examples
The donation of original primary material/tissue	Descriptors Clinical features Genetic features	Donor age, sex, ethnicity/geographical ancestry, tissue type, and source Diseases and phenotypes Mutations, omics, and authenticators (e.g., short tandem repeats)
Manufacturing and cryopreservation	Protocols Process data Batch data	Reprogramming methods Culture protocols (e.g., media, passaging, environment, and bioreactors) and genetic modifications of cells
Cells	Characterisation Genetic features Safety Genetic stability	Cell morphology, differentiation potency (such as teratoma formation or marker expression), passage number, karyotype, omics, authenticators, cell line quality standard (e.g., research vs. clinical use), vector clearance, and microbiology/virus screening
Applications	Drug screening Disease models Clinical	Organoids, differentiated cell types, and clinical trials
Legal and ethics	Patents MTAs Consent documentation templates	Informed consent data Ethics approvals Restrictions for the use of cells and data (e.g., commercial, research, clinical, and global access)

**Table 3:** Infrastructures and resources used by EBiSC. “X” marks where specific management tasks are assigned.

Management Task	hPSCreg^®^	IMS	EBiSC Catalogue	EBI/EGA
Cell line registration by depositors	X			
Unique identifier/standard name through an automated programming interface (API)	X	X		
Standardised iPSC data	X			
Ontology lookup				X
Searchable, publicly accessible database	X		X	
iPSC data validation	X			
Ethics provenance assessment/validation	X			
Genetic data storage and access				X
Data management tools/dashboard		X		
Inventory/batch management		X		
Batch certificate of analysis management		X		
Catalogue	X		X	
Operational alignment, internal data exchange, and transfer between components/banking facilities		X		
Protocols, user guidance, and support			X	

**Table 4:** Implementation of the FAIR principles.

FAIR Principle	Features
Findable	Globally unique and persistent identifiers are assigned to each registered cell line donor and hPSC line upon cell line registration; Batches and vials are similarly assigned unique identifiers; Cell lines are searchable in multiple resources (e.g., Cellosaurus, ICSCB, and WikiData).
Accessible	Each cell line record has a stable URL; Cell line metadata are retrievable by an API (EBiSC IMS with authentication).
Interoperable	Characteristics of the cell line or its donor are recorded using ontology terms, thereby allowing for knowledge representation.
Reusable	The cell line record is crosslinked to external resources, e.g., hPSCreg^®^, Biosamples, ECACC, Cellosaurus, Wikidata, and PubMed.

**Table 5:** Data on EBiSC cell lines.

**A. Basic data for cell line registration**	
Data type	Specific information
Unique cell line identifiers	Standard stem cell nomenclature
Cell line provider	Generator Owner Distributor
Donor information	Donor characteristics: sex, disease status, clinical features, age, and genotype Ethics/consent: consent documentation and provenance has been established Genetic/clinical data: managed access through EBiSC
**B. iPSC Line Data**	
Data type	Specific information
iPSC derivation	Source cell type (donated tissue) Potency Morphology Marker expression
Genetic data	STR profiles HLA typing Karyotype status
Cultivation conditions	Surface coating, passage method, medium, and use of ROCK inhibitor
Sterility	Microbiology/virology screening status
Genetic modification	Confirmation of gene edits (if applicable) Type of modification: transgene expression, gene knock-in/-out, and isogenic modification
Batch information	Growth characteristics, morphology, differentiation potency, and passage number Authentication results and karyotype Cultivation conditions: matrix, culture medium, and O_2_ and CO_2_ concentrations
Supplementary data	Publications iPSC line applications Omics data at the cell line or donor level
Terms and conditions of use	Allowed usage: research/clinical/commercial Third-party obligations Application grade: research/clinical tracking Access to sensitive personal data (e.g., genetic or clinical data) managed by EBiSC DAC
Operational data	Sales inventory Aggregated cell line sales data

**Table 6:** Key information points for EBiSC consent review.

No.	EBiSC Mandatory Item for Consent Documentation Review	Relevant Questionnaire Statements: Ethics/Usage
1	Was participation and sample/data donation voluntary? Yes: deposition in EBiSC possible No: ineligible for deposition in EBiSC	Has informed consent been obtained from the donor of the tissue from which the pluripotent stem cells have been derived? Was the consent voluntarily given? Has the donor been informed that participation will not directly influence their personal treatment? Will the donor expect to receive financial benefit, beyond reasonable expenses, in return for donating the biosample?
2	Was the generation of iPSCs or cell lines explicitly mentioned? Yes: deposition in EBiSC possible No: deposition in EBiSC is still possible on a case-by-case basis	Does consent explicitly allow the derivation of pluripotent stem cells? Does consent expressly prevent the derivation of pluripotent stem cells?
3	Can donated samples and derivatives be shared to other researchers at non-profit and for-profit organisations internationally? Yes: deposition in EBiSC possible No: ineligible for deposition in EBiSC	Does consent prevent the DONATED BIOSAMPLE from being made available to researchers anywhere in the world? Does consent prevent CELLS DERIVED FROM THE DONATED BIOSAMPLE from being made available to researchers anywhere in the world? Does consent permit research by: ∘ an academic institution? ∘ A public organisation? ∘ A non-profit company? ∘ A for profit-corporation?
4	Is the generation, analysis, storage and sharing of genomic data included? Yes: deposition in EBiSC possible No: ineligible for EBiSC deposition	Does consent expressly permit collection of genetic information? Does consent expressly permit storage of genetic information? Does consent prevent dissemination of genetic information? How may genetic information associated with the cell line be accessed?
5	Is undefined ‘future research’ consented for? Yes: deposition in EBiSC possible No: eligibility for deposition in EBiSC is subject to ad hoc ethical review considering the status quo of research at the time of consent vs. present day	Does consent permit unforeseen future research, without further consent?
6	What are the consequences of consent withdrawal? Remark: Discussion with clinical representative to clarify that once cell lines are deposited in EBiSC and distributed, fully halting their use cannot be guaranteed.	Does the consent permit the donor, upon withdrawal of consent, to stop the use of the derived cell line(s) that have already been created from donated samples? Does the consent permit the donor, upon withdrawal of consent, to stop delivery or use of information and data about the donor?
7	Have consent templates undergone independent ethical review according to local procedure? Yes: deposition in EBiSC possible No: ineligible for EBiSC deposition	Has a favourable opinion been obtained from a research ethics committee, or other ethics review panel, in relation to the Research Protocol including the consent provisions?
8	Do consent templates contain restrictions for use of the cell line/derivatives? Yes: these restrictions should be explained explicitly and deposition in EBiSC is possible No: deposition in EBiSC is possible.	Does consent pertain to a specific research project?
9	When did sample collection take place, e.g., before or after GDPR came into effect? Remark: for samples collected after 2018 and in the EU, data protection clarification is mandatory for deposition in EBiSC.	Source cell lot: Year when the source cell was collected
10	Do consent templates prevent or limit potential commercial exploitation of derivatives? Yes: this limitation should be explained explicitly and deposition in EBiSC is possible No: deposition in EBiSC is possible as long as cells can still be used by commercial companies for research purposes.	Does consent expressly prevent development of commercial products? Does consent expressly prevent financial gain from any use of the donated embryo/tissue, including any product made from it?

**Table 7:** Documents and processes related to deposition and use of EBiSC cell lines and data.

**Cells**		
EMDA	EBiSC Material Deposit Agreement	Legal agreement between the depositor and EBiSC to grant EBiSC the right to bank, QC, and distribute a depositor’s cell line
CLIP/PIP	Cell Line or Product Information Pack	Contains all iPSC information relating to permitted and restricted uses (as IP or TPOs)
EAUA	EBiSC Access and Use Agreement	Legal agreement between EBiSC and the user to purchase and use a cell line, including information on existing TPOs
**Data**		
DAC	Data Access Committee	Manages user access to sensitive personal data associated with an EBiSC cell line
DAA	Data Access Agreement	Legal agreement between EBiSC and the user to use sensitive personal data

## Data Availability

Data and more information about EBiSC are available at https://ebisc.org.

## References

[1] Takahashi K., Yamanaka S. (2006). Induction of pluripotent stem cells from mouse embryonic and adult fibroblast cultures by defined factors. Cell.

[2] Kim J.-H., Kawase E., Bharti K., Karnieli O., Arakawa Y., Stacey G. (2022). Perspectives on the cost of goods for hPSC banks for manufacture of cell therapies. NPJ Regen. Med..

[3] Under Development: Biotechnology—Data Interoperability for Stem Cell Data—Part 1: Framework. https://www.iso.org/standard/83185.html.

[4] International Society for Stem Cell Research Guidelines for Stem Cell Research and Clinical Translation 2021. https://www.isscr.org/s/isscr-guidelines-for-stem-cell-research-and-clinical-translation-2021.pdf.

[5] Sakurai K., Kurtz A., Stacey G., Sheldon M., Fujibuchi W. (2016). First Proposal of Minimum Information About a Cellular Assay for Regenerative Medicine. Stem Cells Transl. Med..

[6] de Sousa P.A., Steeg R., Kreisel B., Allsopp T.E. (2017). Hot Start to European Pluripotent Stem Cell Banking. Trends Biotechnol..

[7] Sousa P.A., de Steeg R., Wachter E., Bruce K., King J., Hoeve M., Khadun S., McConnachie G., Holder J., Kurtz A. (2017). Rapid establishment of the European Bank for induced Pluripotent Stem Cells (EBiSC)—the Hot Start experience. Stem Cell Res..

[8] Steeg R., Neubauer J.C., Müller S.C., Ebneth A., Zimmermann H. (2020). The EBiSC iPSC bank for disease studies. Stem Cell Res..

[9] Kurtz A., Mah N., Chen Y., Fuhr A., Kobold S., Seltmann S., Müller S.C. (2022). Human pluripotent stem cell registry: Operations, role and current directions. Cell Prolif..

[10] Mah N., Seltmann S., Aran B., Steeg R., Dewender J., Bultjer N., Veiga A., Stacey G.N., Kurtz A. (2020). Access to stem cell data and registration of pluripotent cell lines: The Human Pluripotent Stem Cell Registry (hPSCreg). Stem Cell Res..

[11] Seltmann S., Lekschas F., Müller R., Stachelscheid H., Bittner M.-S., Zhang W., Kidane L., Seriola A., Veiga A., Stacey G. (2016). hPSCreg—The human pluripotent stem cell registry. Nucleic Acids Res..

[12] Wilkinson M.D., Dumontier M., Aalbersberg I., Jsbrand J., Appleton G., Axton M., Baak A., Blomberg N., Boiten J.-W., da Silva Santos L.B. (2016). The FAIR Guiding Principles for scientific data management and stewardship. Sci. Data.

[13] Kurtz A., Seltmann S., Bairoch A., Bittner M.-S., Bruce K., Capes-Davis A., Clarke L., Crook J.M., Daheron L., Dewender J. (2018). A Standard Nomenclature for Referencing and Authentication of Pluripotent Stem Cells. Stem Cell Rep..

[14] Courtot M., Cherubin L., Faulconbridge A., Vaughan D., Green M., Richardson D., Harrison P., Whetzel P.L., Parkinson H., Burdett T. (2019). BioSamples database: An updated sample metadata hub. Nucleic Acids Res..

[15] Athar A., Füllgrabe A., George N., Iqbal H., Huerta L., Ali A., Snow C., Fonseca N.A., Petryszak R., Papatheodorou I. (2019). ArrayExpress update—From bulk to single-cell expression data. Nucleic Acids Res..

[16] Freeberg M.A., Fromont L.A., D’Altri T., Romero A.F., Ciges J.I., Jene A., Kerry G., Moldes M., Ariosa R., Bahena S. (2022). The European Genome-phenome Archive in 2021. Nucleic Acids Res..

[17] Bairoch A. (2018). The Cellosaurus, a Cell-Line Knowledge Resource. J. Biomol. Tech. JBT.

[18] Steeg R., Mueller S.C., Mah N., Holst B., Cabrera-Socorro A., Stacey G.N., De Sousa P.A., Courtney A., Zimmermann H. (2021). EBiSC best practice: How to ensure optimal generation, qualification, and distribution of iPSC lines. Stem Cell Rep..

[19] Diehl A.D., Meehan T.F., Bradford Y.M., Brush M.H., Dahdul W.M., Dougall D.S., He Y., Osumi-Sutherland D., Ruttenberg A., Sarntivijai S. (2016). The Cell Ontology 2016: Enhanced content, modularization, and ontology interoperability. J. Biomed. Semant..

[20] Mungall C.J., McMurry J.A., Köhler S., Balhoff J.P., Borromeo C., Brush M., Carbon S., Conlin T., Dunn N., Engelstad M. (2017). The Monarch Initiative: An integrative data and analytic platform connecting phenotypes to genotypes across species. Nucleic Acids Res..

[21] Schriml L.M., Arze C., Nadendla S., Chang Y.-W.W., Mazaitis M., Felix V., Feng G., Kibbe W.A. (2012). Disease Ontology: A backbone for disease semantic integration. Nucleic Acids Res..

[22] Vasant D., Chanas L., Malone J., Hanauer M., Olry A., Jupp S., Robinson P.N., Parkinson H., Rath A. ORDO: An Ontology Connecting Rare Disease, Epidemiology and Genetic Data. Proceedings of the 22nd Annual International Conference on Intelligent Systems for Molecular Biology.

[23] Besser D. (2020). Advancing Stem Cell Technologies and Applications: A Special Collection from the PluriCore Network in the German Stem Cell Network (GSCN). Curr. Protoc. Stem Cell Biol..

[24] Dahéron L., Diecke S., Healy L., D’Souza S. (2021). Cores laboratories: Organization for stem cell technology advancement. Stem Cell Res..

[25] Vasilevsky N., Johnson T., Corday K., Torniai C., Brush M., Segerdell E., Wilson M., Shaffer C., Robinson D., Haendel M. (2012). Research resources: Curating the new eagle-i discovery system. Database J. Biol. Databases Curation.

[26] Chen Y., Sakurai K., Maeda S., Masui T., Okano H., Dewender J., Seltmann S., Kurtz A., Masuya H., Nakamura Y. (2021). Integrated Collection of Stem Cell Bank Data, a Data Portal for Standardized Stem Cell Information. Stem Cell Rep..

[27] Kurtz A., Elsallab M., Sanzenbacher R., Abou-El-Enein M. (2019). Linking Scattered Stem Cell-Based Data to Advance Therapeutic Development. Trends Mol. Med..

